# Oxidative Characteristics of Turkey Hemoglobin A Containing Covalently Bound Epigallocatechin Gallate

**DOI:** 10.1021/acs.jafc.5c17482

**Published:** 2026-06-09

**Authors:** Jie Yin, Wenjing Zhang, Nantawat Tatiyaborworntham, Craig A. Bingman, Mark P. Richards

**Affiliations:** † Meat Science and Animal Biologics Discovery, Animal and Dairy Sciences Department, 5228 University of Wisconsin-Madison, Madison, Wisconsin 53706, United States; ‡ Biochemistry Department, 5228 University of Wisconsin-Madison, Madison, Wisconsin 53706, United States

**Keywords:** heme proteins, oxidative rancidity, thiol, quinone, quality deterioration; lipid oxidation, protein crystallography, turkey

## Abstract

We investigated the binding of epigallocatechin gallate (EGCG) to turkey hemoglobin A (Hb), noting that polyphenols have the capacity to inhibit oxidative deterioration in muscle foods mediated by endogenous hemoglobin. The addition of EGCG to MetHb resulted in covalently bound EGCG to Cys^130^ of both α-chains. The crystal structure showed that each bound EGCG was located near the other and in the protein interior. Distances between the nearest phenol/phenolate of bound EGCG and the nearest iron atom of the heme moieties were 11.7–16.5 Å. Antioxidative characteristics due to bound EGCG included decreases in both hemin dissociation and H_2_O_2_-mediated ferryl Hb formation, counterbalanced by increased Hb autoxidation. Bound EGCG less effectively inhibited oxyHb-mediated lipid oxidation compared to MetHb-mediated lipid oxidation. The mechanisms by which EGCG adduction affected oxidative characteristics of Hb are discussed, including electron transfer from bound EGCG to the heme, interactions with lipids, and effects of cross-linking on hemin affinity.

## Graphical abstract

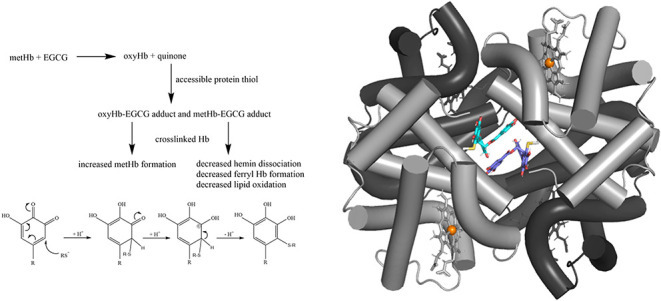

## Introduction

Lipid oxidation during the storage of muscle foods leads to off-odors and off-flavors that contribute to food waste. Lipid oxidation is noted to occur rapidly in turkey muscle, attributable to the pro-oxidative capacity of hemoglobin (Hb).^1,2^ The addition of polyphenols to counteract turkey Hb is one approach to decreasing lipid oxidation, yet the mechanisms of antioxidant action by polyphenols are not fully understood. Polyphenols can inhibit lipid oxidation by free-radical scavenging and chelation of metals.^3^ Another means by which polyphenols can affect oxidative processes is through covalent bonding to proteins when a catechol, pyrogallol, or galloyl unit is part of the structure.^4^ The pyrogallol upon donation of two electrons is converted to a quinone (Figure 1). The electrophilic quinone is reactive with nucleophiles, such as sulfhydryl groups of proteins, to form covalent adducts in which the bound quinone is reduced to its pyrogallol form (Figure 1). Hemoglobin (Hb) from mammals, birds, and fish contains free sulfhydryl groups, which suggests that pyrogallol-, galloyl-, and catechol-containing polyphenols can covalently bind at these sites to modify Hb.^5–7^ Covalent binding of small molecules to Hb has the potential to alter the oxidative properties of the heme protein. For example, covalent binding of 4-hydroxy-2-nonenal to histidine residues of myoglobin (Mb) has been shown to increase rates of metMb formation.^8^ Covalent bonding of cysteine to the catechol unit-containing caffeic acid (CA) was reported to decrease metMb formation more effectively than CA alone, suggesting that CA-bound thiols in proteins may be particularly effective in stabilizing red color in raw muscle foods.^9^ CA inhibited lipid oxidation in minced, mackerel muscle more effectively compared to other hydroxycinnamic acids that lacked a catechol unit, suggesting that the catechol may be a key functional group by which lipid oxidation is inhibited.^10^ Galloylated polyphenols were noted to inhibit lipid oxidation in mackerel muscle more effectively than nongalloylated polyphenols.^11^ Noncovalent interactions of polyphenols with bovine Hb were mainly associated with the hydrophobic force effect.^12^ Multispectroscopic and simulation studies that examined binding of rutin and quercetin to bovine hemoglobin were noted to not disrupt secondary structure, while epigallocatechin gallate (EGCG) binding decreased α-helical content.^13,14^ Hydrogen bonding and van der Waals forces were attributed to facilitate noncovalent binding of rosmarinic acid and kaempferol to grass carp hemoglobin.^15^ Further studies are needed to characterize aspects of the interaction between Hb and polyphenols, notably whether covalent bonding takes place. It should also be noted that these previous reports describe mammalian and fish Hbs, while investigation of polyphenol interaction with avian hemoglobins is lacking.

**Figure 1:**
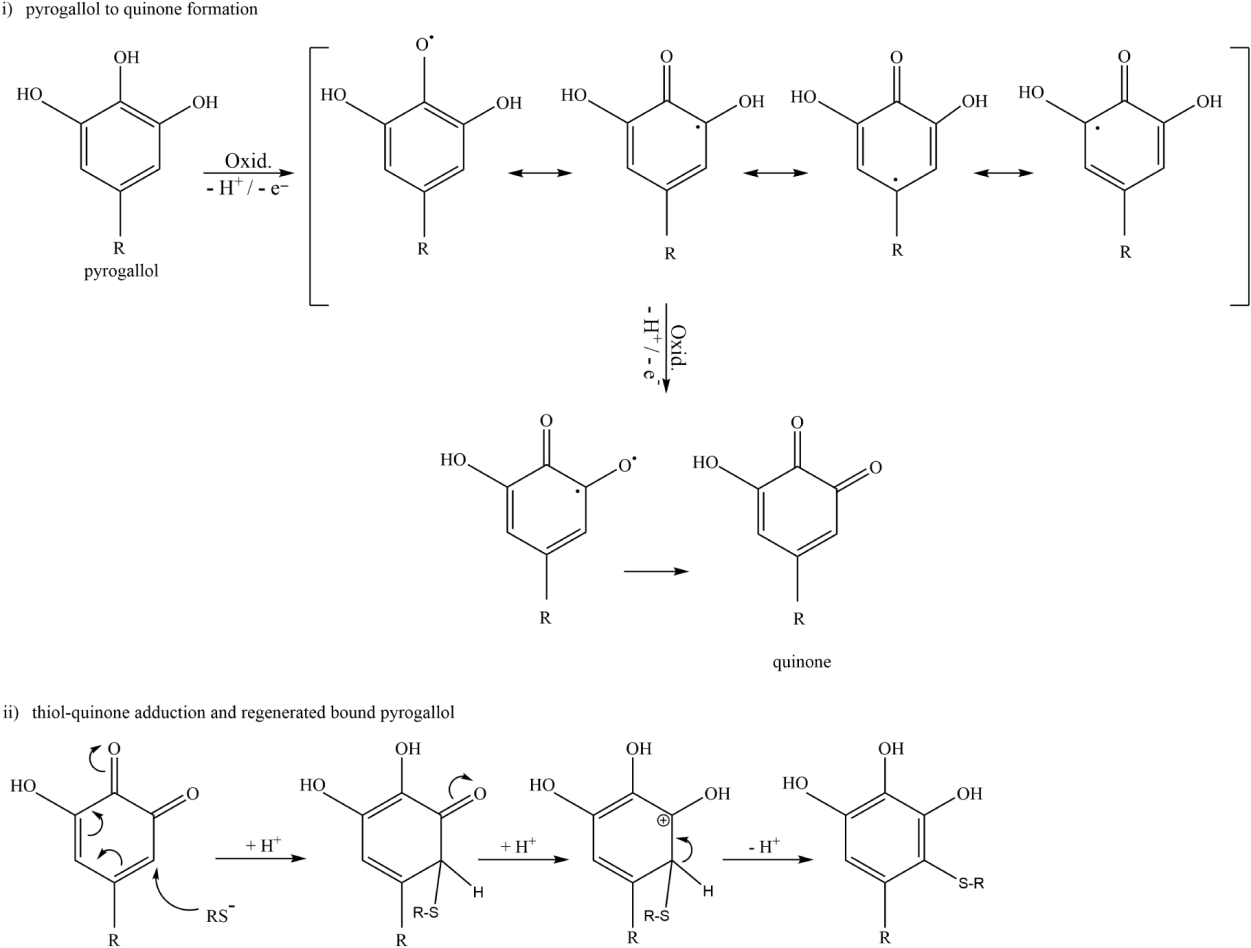
Formation of a covalent adduct from the reaction of quinone with a protein containing an available sulfhydryl side chain. (i) Quinone formation; (ii) formation of adducted protein. Adapted with permission from ref 4. Copyright 1988 Elsevier.

Hemoglobin (Hb) is a tetrameric protein comprised of four distinct subunits, namely α_1_, α_2_, β_1_, and β_2_, with each chain containing a protoporphyrin IX (PPIX) moiety that has a central iron atom (Fe^2+^-PPIX in oxyHb and deoxyHb, Fe^3+^-PPIX in MetHb, and Fe^4+^-PPIX in ferryl Hb). MetHb oxidized lipids in washed muscle more readily than oxyHb,^16^ whereas oxyHb promoted lipid oxidation more readily than MetHb in linoleic acid micelles with a low lipid hydroperoxide (LOOH) content.^17^ Hydrogen peroxide (H_2_O_2_) is generated in biological tissues by various pathways, including Hb autoxidation.^18^ H_2_O_2_ reacts with deoxyHb and MetHb to form ferryl Hb and perferryl Hb radical, respectively.^19^ Ferryl Hb incurs lipid oxidation upon reaction with H_2_O_2_ or LOOH, while perferryl Hb radical can initiate lipid oxidation through abstraction of a hydrogen atom from a polyunsaturated fatty acid.^19^ Alternatively, the perferryl Hb radical can cause cross-linking within Hb and with other proteins.^20^ MetHb formed after Hb autoxidation can also release Fe^3+^-PPIX (termed hemin) that effectively oxidizes lipids through the decomposition of preformed LOOH.^21^ The objective of this work was to investigate binding of EGCG to Hb and associated oxidative effects toward Hb and lipids. EGCG was selected for investigation, noting it is the most abundant catechin in green tea leaves (∼59% of total catechins), with the catechins comprising 25–35% of the dry weight.^22,23^ Green tea extracts are increasingly utilized to inhibit lipid oxidation in muscle foods, including those containing turkey muscle.^22,24,25^ A better understanding of how EGCG modulates the pro-oxidative activity of turkey Hb can be used to further develop green tea extracts for application in muscle foods. Protein crystallography was utilized to investigate the location of bound polyphenol.

## Materials and Methods

### Chemicals

Epigallocatechin-3-gallate (EGCG), catalase (from bovine liver), superoxide dismutase (SOD, from bovine erythrocyte), ferric chloride, ferrous sulfate, disodium ethylenediaminetetraacetic acid (EDTA) dihydrate, potassium ferricyanide, streptomycin sulfate, barium chloride, trichloroacetic acid (TCA), 2-thiobarbituric acid (TBA), and ammonium thiocyanate were obtained from Sigma Chemical A/S (St. Louis, MO). Chloroform, methanol, ethanol, sodium chloride (NaCl), and tris­(hydroxymethyl)­aminomethane (Tris) were obtained from Fisher Scientific (Pittsburgh, PA). Sodium dodecyl sulfate (SDS) was purchased from Hoefer Inc. (San Francisco, CA). All other chemicals used were of analytical grade.

### Preparation of Turkey HbA

Whole blood from 5- to 14-week-old turkeys (*Meleagris gallopavo*) was obtained with heparin anticoagulant (95 U/mL) in 0.9% NaCl. Procedures to obtain whole blood were approved by the Institutional Animal Care and Use Committee at the University of Wisconsin–Madison under case number A005420. The hemolysate was then prepared as previously described.^2^ The hemolysate was purified using an LCC-501 Plus FPLC system equipped with a HiLoad gel filtration column containing Superdex 200 (GE Healthcare Inc., Uppsala, Sweden) to remove contaminants (column dimensions: 2.6 cm × 60 cm). The hemolysate (6 mL) in 10 mM Tris (pH 8.0), 50 mM NaCl was passed through the column at a flow rate of 2 mL/min using an elution buffer of 10 mM Tris (pH 8.0), 50 mM NaCl. Electrospray ionization mass spectrometry (ESI-MS) was utilized and indicated only α and β chains of hemoglobin-A (HbA) and hemoglobin-D (HbD) were present. HbA was separated from HbD using DEAE-cellulose.^26^ The major component HbA was eluted with the elution buffer of 20 mM Tris, pH 8.5, 1 mM EDTA. HbA was frozen as 30 μL beads using liquid nitrogen and stored at −80 °C. The purified HbA was buffer-exchanged into 1 mM Tris (pH 8.0) using a PD-10 column prior to use. The HbA concentration on a heme basis was measured spectrophotometrically using the millimolar extinction coefficient of 125 mM^–1^ cm^–1^ at 415 nm for oxyHb.^27^ Turkeys have two distinct α-globin genes, resulting in HbA (α^A^_2_β_2_) and HbD (α^D^_2_β_2_) at a 3:1 ratio in turkey blood.^28^ HbA was used for all experiments.

### Preparation of Methemoglobin (MetHb)

MetHb was prepared as described previously.^29^ Briefly, MetHb on a heme basis was reacted with potassium ferricyanide (1:3 molar ratio) for 1 h at 2 °C, followed by the removal of ferricyanide with a PD-10 column equilibrated with 1 mM Tris (pH 8.0). Ferricyanide converts reduced Hb to MetHb.

### Reaction of MetHb with EGCG and Removal of Unreacted EGCG

Reaction of MetHb with EGCG was done at pH 8.0 in 10 mM Tris for 48 h at 2 °C. Unreacted EGCG was removed using an LCC-501 Plus FPLC system (Pharmacia Biotech Inc., Uppsala, Sweden) equipped with a HiPrep 26/10 desalting column (GE Healthcare Inc., Uppsala, Sweden) and an elution buffer of 10 mM Tris (pH 8.0) and 50 mM NaCl. Ratios of 1:1, 2:1, and 3:1 of EGCG to MetHb (heme basis) were examined.

### Electrospray Ionization Mass Spectrometry (ESI-MS)

ESI-MS was performed at the Biotechnology Center (University of Wisconsin–Madison). Analyses were carried out on a 6200 LC/MSD-TOF mass spectrometer (Agilent, Palo Alto, CA) with the sample injections as a 10 μL aliquot of 50 μL volume from acetone-precipitated (4:1 vol:vol) protein preparation solubilized in 100% formic acid and subsequently diluted to 5% formic acid:45% water:50% methanol at a 30 μL/ml flow rate from a 4.6 mm diameter autosyringe delivery system (Harvard Apparatus, Holliston, Massachusetts) in 50:50% methanol:water. The following instrumental parameters were used to generate the most optimal protonated ion species [M + H^⊕^] in positive mode: capillary voltage 3500 V; drying gas 6.0 L/min; nebulizer 20 psig; gas temperature 325 °C; Oct DC1 39.5 V; fragmentor 180 V; Oct RF 250 V; Skimmer 60 V. Agilent BioConfirm Software (version A.02.00) was used for data analysis.

### Protein Crystallography

Crystals of Hb and Hb in the presence of EGCG were grown in MRC 2-drop plates using a TTP Labtech Mosquito robot. General screens were conducted at 4 and 20 °C and included Index HT (Hampton Research), JCSG-plus (Molecular Dimensions), and Morpheus (MRC-LMB). Optimal native crystals were obtained from 200 nL HbA stock at 18.7 mg/mL in 10 mM Tris pH 8.0 buffer, mixed with 200 nL of reservoir solution composed of 16% PEG 3350, 0.2 M sodium citrate, and equilibrated against 50 μL of reservoir solution. Crystals were cryoprotected by soaking in a solution made by supplementing 16 μL of reservoir with 2.3 mg solid polyethylene glycol (PEG) 3350. Crystals were looped and cooled by direct immersion in liquid nitrogen. Data were collected at APS Beamline 23ID-B on 2015-04-12 using 0.9793 Å X-rays on a MARMOSAIC 300 mm CCD detector, 300 frames, 0.5 degrees per frame. Optimal crystals of the EGCG adduct were grown by mixing a 200 nL protein stock solution containing 16 mg/mL turkey HbA in 10 mM Tris pH 8.0. 200 nL with 150 nL of reservoir solution composed of 20% PEG 3350, 0.1 M racemic malic acid, 50 mM HEPES pH 7.0, and equilibrating against 50 μL of reservoir in an SD2 plate. Crystals were cryoprotected with a reservoir solution supplemented with additional PEG 3350 to a final concentration of 30%. Crystals of both native HbA and EGCG-HbA were looped from the cryoprotectant solution and cooled by direct immersion in liquid nitrogen. Native Hb data were collected at APS Beamline 23ID-D on 2015-04-12 using 0.9793 Å X-rays on a MARMOSAIC 300 mm CCD detector. HbA-EGCG data were collected at APS Beamline 23ID-B on 2017-06-14, using a Dectris Eiger X 16 M detector, 1.0332 Å X-rays, 1440 frames, 0.25°. Diffraction data were reduced using XDS.^30^ The native structure was solved by molecular replacement starting with PDB 3K8B.^31^ The EGCG structure was solved using our native structure as the starting model. The structure was alternatively refined in phenix.refine, manually fitted using COOT, and validated using MolProbity.^32,33^ Software used in this study was installed and configured by SBGrid.^34^ The PDB IDs for MetHbA and MetHbA-EGCG are 9O84 and 9O72, respectively. The data collection and refinement statistics for both structures can be found in Table S1 of Supporting Information.

### Hb Autoxidation (MetHb Formation)

When partially oxidized Hb was obtained, anaerobic reduction was done as previously described.^35^ The concentration of Hb and the rates at which reduced Hb autooxidized to MetHb were determined at pH 5.7 and 25 °C (200 mM bis-Tris) using the equations based on the absorbance changes at 630, 576, and 560 nm as described previously.^36,37^ The autoxidation rate (*k*_ox_) was calculated by curve fittings using an exponential function based on the normalized data with the use of superoxide dismutase and catalase to inhibit ferryl Hb formation during storage. Protein concentration as described previously^38^ was also determined to ensure that adducted and nonadducted Hb were examined at equivalent concentrations for Hb autoxidation, hemin dissociation, ferryl Hb formation, and lipid oxidation capacity measurements.

### Dissociation of Hemin (Fe^3+^-Protoporphyrin IX) from MetHb

Dissociation of hemin from MetHb was determined at pH 6.3 and 25 °C using the apoH64Y/V68F Mb reagent.^39^ Upon transfer of hemin from MetHb into apoH64Y/V68F, the optical density at 600 nm increases, which was quantified spectrophotometrically.

### H_2_O_2_-Mediated Ferryl Hb Formation

MetHbA and MetHbA-EGCG adduct (30 μM on a heme basis) were reacted with H_2_O_2_ (30 and 7.5 μM) for 5 min at 25 °C (pH 7.4) before adding sodium sulfide (Na_2_S) to convert ferryl heme into sulfheme that was monitored by the optical density peak at 618 nm.^40^ The concentration of sulfheme was calculated using the extinction coefficient ε_618 nm_ = 21.1 mM^–1^ cm^–1^.

### SDS Polyacrylamide Gel Electrophoresis (SDS-PAGE)

Hb was added to 100 μL of a sample buffer (20 mL of glycerol, 40 mL of 10% SDS, 25 mL of 0.5 M Tris buffer at pH 6.8, and 15 mL of Milli-Q water) with 10% β-MeSH and incubated for 5 min at 95 °C. The prepared samples (2.0 mg protein/mL) were loaded at 15 μg on a 3% polyacrylamide stacking gel and a 12% polyacrylamide resolving gel. The loaded gel was run at a constant 120 V (with a running time of approximately 2.5 h). The protein bands were stained with a staining solution (1 mg/mL of Coomassie Brilliant Blue R-250 in 50% methanol and 6.8% acetic acid) for 3 h with constant shaking at 70 rpm. The stained gels were destained with a destaining solution (7.5% acetic acid and 5% methanol in Milli-Q water) overnight. Afterward, the gels were scanned at 600 dpi resolution in the JPEG image format. Gel images were processed with Adobe Photoshop CS5.1 to obtain the best contrast for densitometry analysis through software.

### Native Polyacrylamide Gel Electrophoresis (Native-PAGE)

Native-PAGE was carried out by PhastSystem automated flatbed electrophoresis units (Amersham Bioscience Co., Piscataway, NJ). In brief, electrophoretic analyses were performed on 4–15% polyacrylamide gradient gels (GE Healthcare Inc., Uppsala, Sweden) at a constant voltage (400 V) and at 15 °C, employing the PhastGel buffer strips containing 0.88 M l-alanine and 0.25 M Tris at pH 8.8 to create a region of uniform voltage and constant pH. Proteins (∼1 mM, ∼1 μL) were loaded on the gradient gels, and electrophoresis was run for 30–40 min until the assayed sample reached the end of the gel. Gels were stained with Bio-Safe Coomassie Blue (G-250) (Bio-Rad Inc., Hercules, CA) overnight on a shaker. Gels were destained directly by rinsing in Milli-Q water and then scanned at 600 dpi resolution in the JPEG image format. Gel images were processed with Adobe Photoshop CS5.1 to obtain the best contrast for densitometry analysis through software.

### Preparation of Washed Cod Muscle (WCM)

Fillets from Atlantic cod (*Gadus morhua*) were obtained by overnight delivery. The fillets were never frozen. The washed cod muscle (WCM) was prepared as described previously and stored at −80 °C until use.^41^ Concern regarding residual heme protein after washing is mostly avoided in WCM, noting the very low heme protein content in the cod muscle starting material.

### Addition of Hb to WCM

WCM was thawed at room temperature for 15–20 min and transferred to a plastic beaker on ice and mixed with a plastic spatula for 10 min to break up the pieces of mince. The pH of the muscle was adjusted by 1 N HCl before adding to a 30-ml amber bottle. Water was added to the vial to obtain a final moisture content of 90%, and the streptomycin sulfate stock (2% w/v) was added to the washed mince at a final concentration of 200 ppm to inhibit microbial growth. The heme proteins were added into WCM to obtain a final concentration of 40 μmol/kg WCM (heme basis). The contents of the vial were mixed thoroughly with a plastic spatula for 2 min. Samples were taken at 0 h of iced storage for analysis.

### Determination of Lipid Peroxides

Lipid peroxides were measured as described previously.^42,43^ A standard curve was constructed using cumene hydroperoxide. Lipid peroxides were expressed as μmol/kg WCM (wet weight).

### Determination of Thiobarbituric Acid Reactive Substances (TBARS)

TBARS were determined as described previously.^44^ A standard curve was constructed using tetraethoxypropane. TBARS were expressed as μmol TBARS/kg WCM (wet weight).

### Statistical Analysis

Data were analyzed using the SAS program (ver. 9.4). The PROC MIXED procedure was used to analyze the data from the storage studies. For each treatment, two or three separate reactions were examined at each time point during storage. Means were separated by using the *p*-diff test. Significance was determined using a *p* value of less than 0.05.

## Results

### Adduction of EGCG to Turkey MetHb Based on ESI-MS

MetHbA was reacted with equimolar EGCG for 48 h at 2 °C (pH 8.0), followed by ESI-MS to determine the formation of covalent adduct(s). Monoadduction of EGCG to the α-chain of Hb was observed based on the detected mass of 15 793 Da, relative to the mass of 15 338 Da for native Hb α-chain (Figure 2). The 454 *m*/*z* difference before and after reaction with EGCG corresponds to one molecule of EGCG quinone covalently bound to the α-chain. The content of nonadducted α-chain (15 338 Da) was negligible after reaction with EGCG (Figure 2). This indicated that nearly all the α-chains of MetHb exposed to EGCG contained the monoadduct. No adduction on the Hb β-chain by EGCG was observed; the *m*/*z* of 16,307 is indicative of the native β-chain (Figure 2). An increase of 98 Da was observed on the α- and β-chains of native HbA, as well as the adducted α-chain of HbA. This has been attributed to the binding of one sodium and two potassium ions to proteins.^45^ Since ESI-MS does not provide the location where EGCG became covalently bound, protein crystallography was used to ascertain the location as described below. It is acknowledged that pH 8.0 used to react EGCG with MetHb is elevated compared to postmortem muscle tissue and physiological conditions. At the same time, we have observed that polyphenols do covalently bind to turkey HbA α-chains at pH values as low 6.0, but with heterogeneity, such as incomplete adduction of the α-chains (manuscript in preparation). The likelihood of obtaining a high-resolution crystal structure diminishes when there is a mixed population of adducted and nonadducted α-chains. Having the α-chains fully adducted with EGCG as occurred at pH 8.0 (Figure 2) provided an opportunity to solve the crystal structure to determine the site(s) where EGCG became covalently bound.

**Figure 2:**
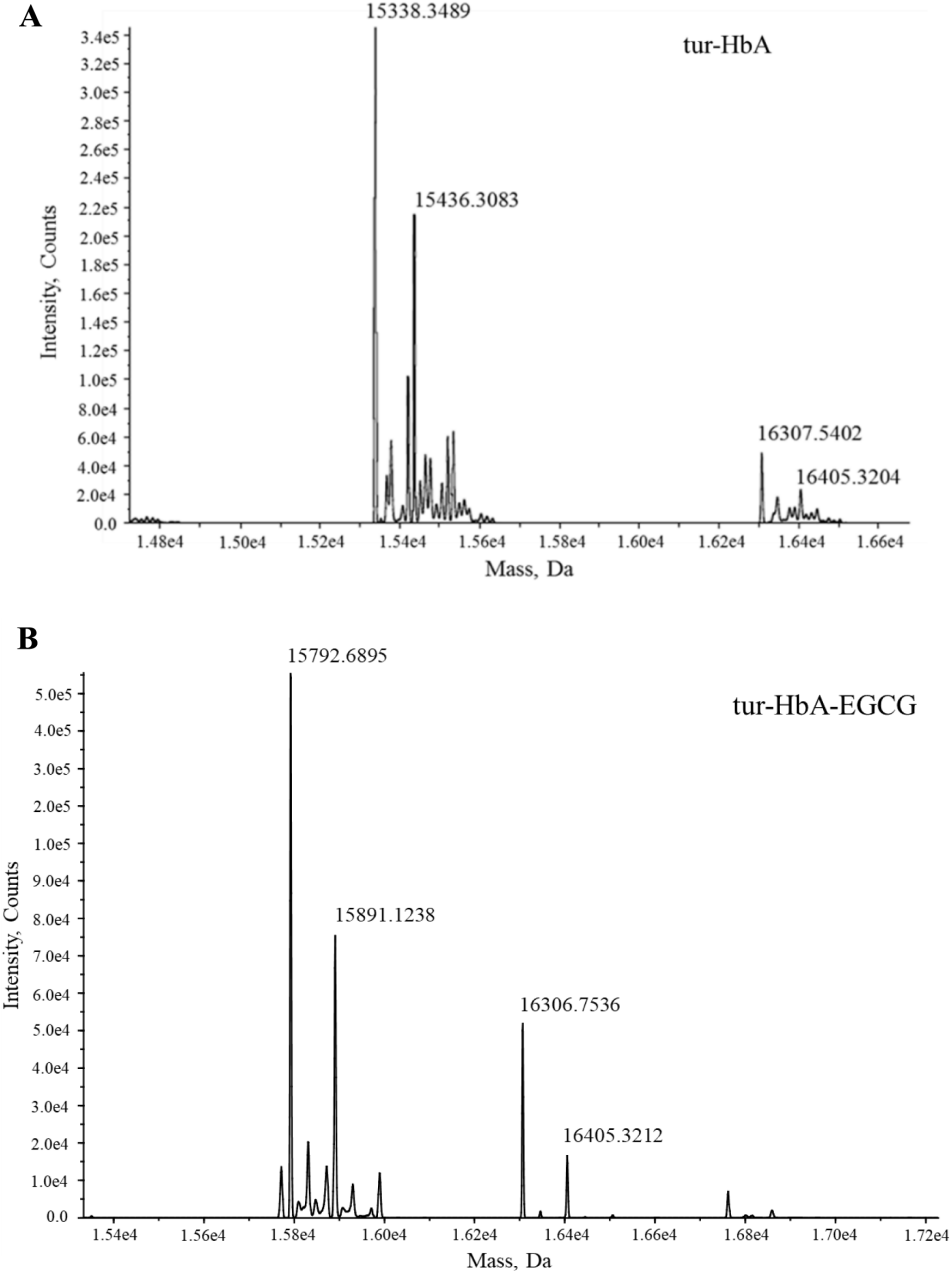
Electrospray ionization mass spectrometry (ESI-MS) spectra. (A) Turkey MetHbA; (B) MetHbA exposed to EGCG for 48 h at pH 8.0 and 2 °C. 0.5 mM MetHbA (heme basis) reacted with 0.5 mM EGCG.

### Crystallographic Analysis of Turkey MetHbA-EGCG

After initial screenings and further optimizations, HbA containing the monoadduct of EGCG on the α-chain was examined by X-ray diffraction. The crystals belonged to space group P 21 21 21 and diffracted to 1.99 Å resolution, and the positions of EGCG bound to HbA were unambiguous. A single covalent adduction of EGCG occurred with the cysteine residue at site H13 (residue 130) in each α-chain (Figure 3A and B). The sulfhydryl group of Cys was the site for interaction with the 2′C on the B-ring of EGCG. The structure is termed HbA-Cys^130α‑EGCG^ and abbreviated as Hb-EGCG. For the EGCG bound to one α-chain, the esterified galloyl unit was mostly parallel to and ∼4 Å from the esterified galloyl unit of EGCG bound to the other α-chain (Figure 3B). EGCG bound at Cys^130α^ was mostly buried within the interior of the tetramer (Figure 3B). The distance from the nearest phenol groups of EGCG to the nearest iron atom of the heme moieties within α- and β-chains was 11.7–12.1 Å and 16.3–16.5 Å, respectively (Figure 3C).

**Figure 3:**
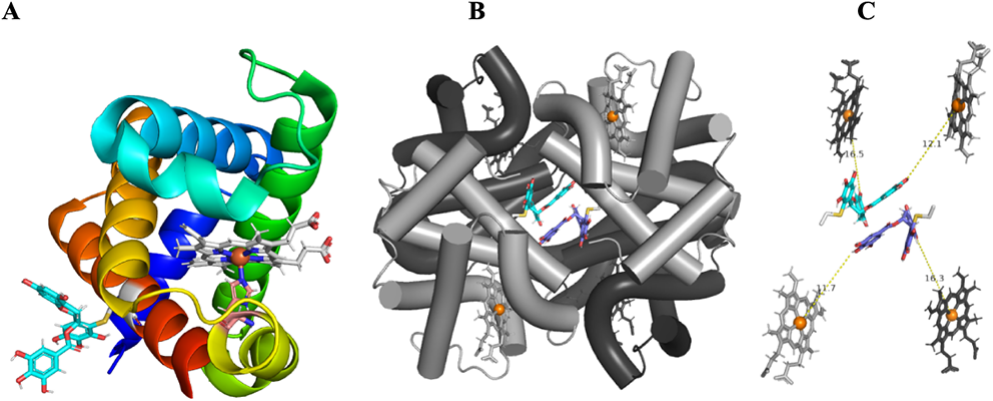
Epigallocatechin gallate (EGCG) bound to turkey HbA at site H13 of α-chains. (A) EGCG covalently bound to Cys^130^ (site H13) in the H-helix of the α-chain^A^ of turkey HbA. The heme moiety, Cys^130^, bound EGCG, and the proximal histidine (site F8) bonding with the iron atom of the heme moiety are shown in stick representation (carbon atoms of EGCG, His­(F8), and heme moiety are shown in cyan, pink, and gray, respectively; O atoms in red, N atoms in blue, S of Cys^130^ in maize, and Fe of the heme moiety in orange as a sphere). Each helix is a different color. (B) Tetrameric view of turkey HbA containing one molecule of EGCG bound to Cys^130^ in each α-chain (α-chains in light gray and β-chains in dark gray). Each helix is shown as a cylinder. His­(F8) in each chain is shown in line representation. (C) Distances from the closest electron-donating group of bound EGCG to the closest heme iron atoms of turkey HbA. Carbon atoms of EGCG bound to the α-chain^A^ and α-chain^C^ are shown in cyan and purple, respectively.

### Crystallographic Analysis of Turkey MetHbA

A structural determination of unmodified turkey MetHbA was also done for detailed comparisons. The crystals belonged to space group P 42 21 2 and diffracted to a resolution of 1.69 Å. Structural alignments with native HbA and HbA-Cys^130α‑EGCG^ indicated no detectable structural differences at sites His^45α^(CE3), Lys^61α^(E10), Val^62α^(E11), Ala^65α^(E14), Ser^44β^(CD3) Lys^66β^(E10), Val^67β^(E11), and Ser^70β^(E14), which were previously described to impact autoxidation and hemin loss rates based on structural differences between fish and mammalian Hbs.^5^ The β-chain^B^ and β-chain^D^ of native HbA were indistinct from each other based on the X-ray diffraction data so that structural attributes and the assignment of distances between atoms are represented in one β-chain for the native HbA. α-chain^A^ and α-chain^C^ of native HbA were also indistinct and are represented in one α-chain. Valine was observed at position 70 of the α-chain. Alanine has been described at this position of turkey Hb α-chain in other studies.^6,46^

### Autooxidation of Hb Compared to Hb Adducted with EGCG

The observation that EGCG became covalently bound to α-chains of HbA raised the question of whether the functional properties of Hb could be modulated, including autoxidation in which reduced forms of Hb, oxyHb and deoxyHb, oxidize to MetHb. After the reaction of EGCG with MetHb, the EGCG-adducted Hb was a mixture of MetHb and oxyHb, necessitating reduction with dithionite to obtain fully reduced Hb-EGCG. The resulting reduced Hb-EGCG was then thoroughly desalted to remove the dithionite reductant and any traces of unbound EGCG. Fully reduced forms of Hb and Hb-EGCG are needed to comparatively assess MetHb formation during the storage period. Hb adducted with EGCG autooxidized to MetHb more rapidly compared to Hb (*p* < 0.05). It took ∼7 h for half of Hb-EGCG to be converted to MetHb compared to ∼23 h for nonadducted Hb (Figure 4A).

**Figure 4:**
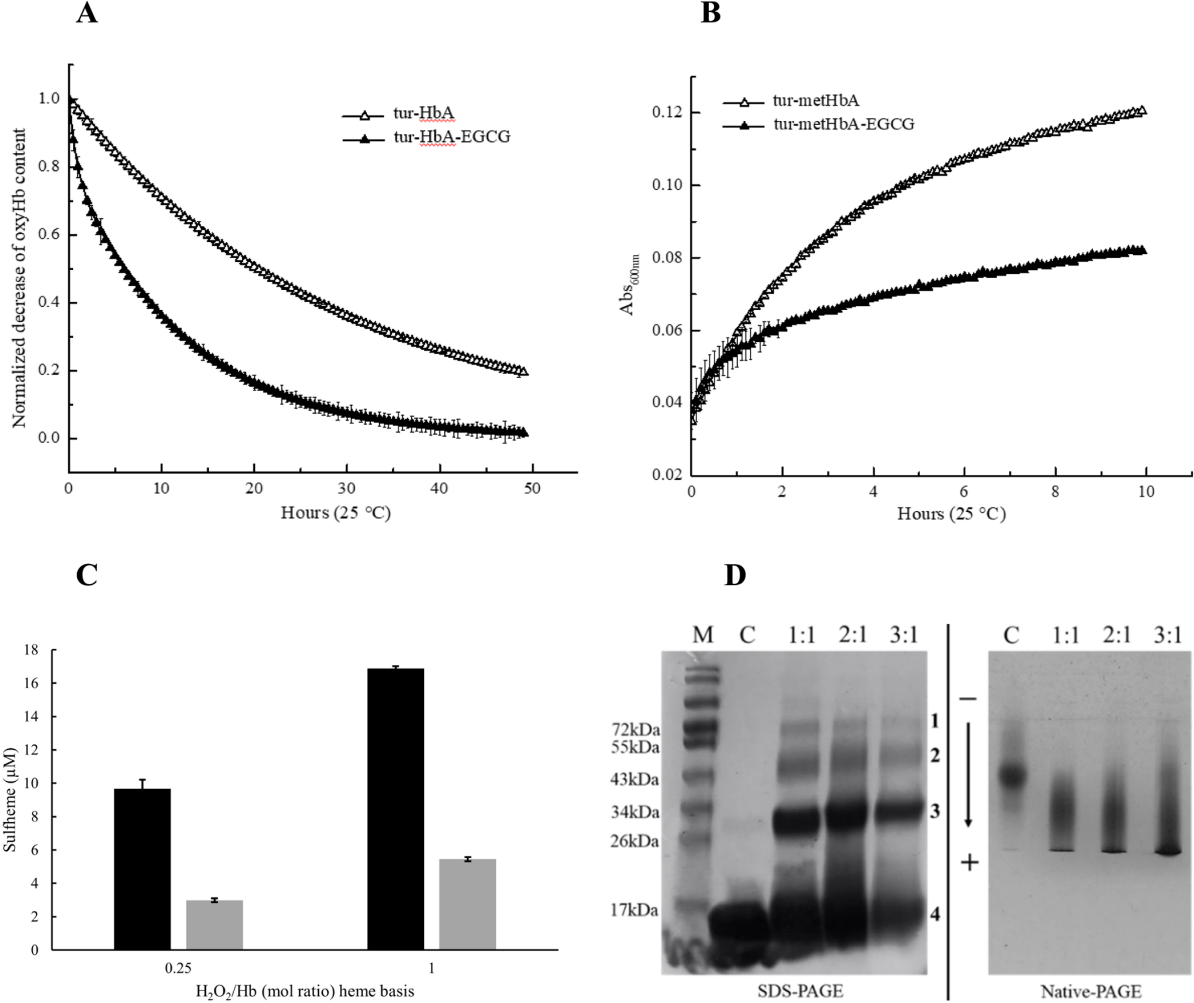
(A) Hb autoxidation of oxyHb vs oxyHb-EGCG (pH 5.7 and 25 °C); (B) hemin loss from MetHb vs MetHb-EGCG (pH 6.3 and 25 °C); (C) H_2_O_2_-mediated ferryl Hb formation of MetHb (black bars) vs MetHb-EGCG (gray bars) at pH 7.4 and 25 °C; (D) crosslinking status based on SDS-PAGE (left) and electrostatic character of MetHb vs MetHb-EGCG using native electrophoresis (right). Ratios shown are that of EGCG to Hb (heme basis). Turkey HbA was used for all measurements.

### Dissociation of Hemin from MetHb Compared to MetHb Containing EGCG Adduct

It was considered that covalently bound EGCG could also affect the release of hemin from Hb, which can accelerate lipid oxidation, noting the affinity of released hemin for phospholipids of cellular membranes.^47^ Since Hb containing adducted EGCG was a mixture of oxyHb and MetHb, potassium ferricyanide was added to obtain MetHb-EGCG to be compared to MetHbA in the hemin dissociation assay. The ferricyanide and unbound EGCG were removed by thorough desalting. It was imperative to ensure both heme proteins were in their MetHb forms since reduced forms of Hb exhibit negligible heme release.^48^ Hemin loss from turkey MetHb was greater compared to MetHb adducted with EGCG (*p* < 0.05). Hemin loss from MetHb-EGCG was approximately half of that from MetHb (Figure 4B).

### H_2_O_2_-Mediated Ferryl Hb Formation in Native MetHbs and MetHbs Adducted with EGCG

EGCG-bound Hb has the potential to reduce hypervalent forms of Hb, such as ferryl Hb and ferryl Hb radical species, noted to be pro-oxidative toward lipids and proteins.^19^ The reaction of H_2_O_2_ with MetHbA and MetHbA-EGCG was for 5 min before adding sodium sulfide (Na_2_S) to convert ferryl heme into sulfheme that could be quantified by the optical density peak at 618 nm. MetHb-EGCG had a decreased ferryl Hb content relative to MetHbA when the molar ratio of H_2_O_2_ to the heme of Hb was 0.25 and 1.0 (Figure 4C). Previous work showed that caffeic acid adducted to sickle cell HbS had decreased ferryl Hb content compared with HbS.^49^

### Effect of EGCG Adduction on the Electrostatic Charge and Polymerization Status of MetHb

Covalently bound EGCG has the potential to alter the net charge of Hb and its interaction with other biomolecules, noting that the various phenol/phenolate groups of EGCG in the protein interior of Hb will be neutral when protonated and negatively charged when unprotonated. Native PAGE results showed that MetHbA-EGCG migrated further toward the positive pole than MetHbA, indicating that the net negative charge on MetHbA increased upon binding of EGCG (Figure 4D). Migration of CO-HbA was compared to MetHbA to determine if there were migration differences between reduced and oxidized Hb. Migration distances were similar when comparing CO-HbA to MetHbA, and it was observed that CO remained bound to HbA during Native PAGE (data not shown). SDS-PAGE was conducted to examine if EGCG incurred polymerization of Hb. Hb as a control exhibited a major band at 17 kDa, representative of the α and β chains comigrating together with no polymerization (Figure 4D). Addition of EGCG caused the formation of a major band in the vicinity of the 34 kDa molecular weight marker. This is consistent with the formation of an Hb dimer. It can be noted that a green tea extract rich in EGCG was described to cross-link proteins via available thiols in a bologna-type sausage from oxidatively stressed pigs.^50^

### Lipid Oxidation due to Hb Compared to Hb Adducted with EGCG

It was of interest to investigate lipid oxidation due to Hb-EGCG compared to Hb since both pro-oxidative and antioxidative effects of bound EGCG were noted from the assays of autoxidation, hemin dissociation, and ferryl Hb formation. The ability of HbA and HbA-EGCG (reduced forms of each heme protein) to promote lipid oxidation was determined in washed muscle during 2 °C storage. The lag phase prior to TBARS formation was similar in HbA and HbA-EGCG, with TBARS values increasing at day 0.75 for each form of oxidant. However, at days 1.5, 2.0, 2.5, and 3, TBARS values were significantly lower in the HbA-EGCG treatment (Figure 6). Maximal TBARS values of 103 and 78 μmol/kg were observed for HbA and HbA-EGCG, respectively (Figure 5).

**Figure 5:**
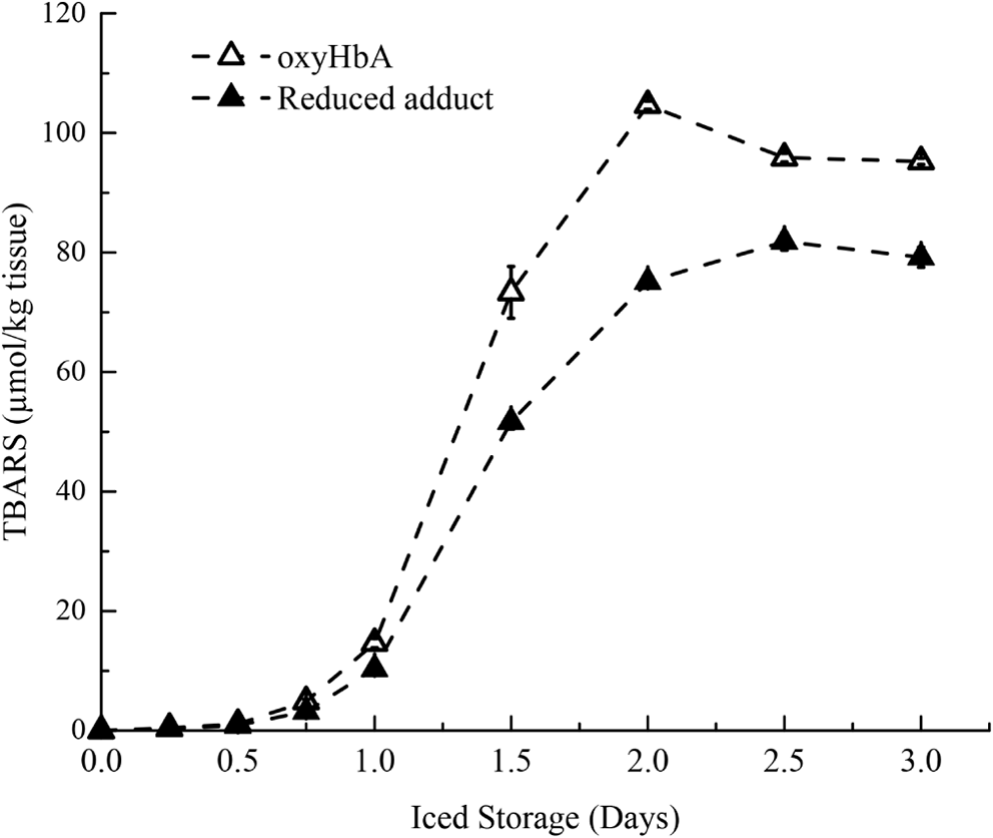
Thiobarbituric acid reactive substances (TBARS) values of oxyHbA and oxyHbA-EGCG adduct in washed muscle during 2 °C storage. pH was 6.3. Hb concentration was 30 μmol/kg washed cod (heme basis).

### Lipid Oxidation due to MetHb Compared to MetHb Adducted with EGCG

The ability of MetHb and MetHbA-EGCG to promote lipid oxidation was also examined. Lipid peroxides and thiobarbituric acid-reactive substances (TBARS) were determined in washed muscle as markers of primary and secondary lipid oxidation products, respectively. Adduction of EGCG to MetHbA resulted in a considerable delay in lipid peroxide formation compared with MetHbA (Figure 6A). Further, the extent of lipid peroxide formation was lower in MetHbA-EGCG compared to that in MetHbA. Regarding TBARS, MetHbA-EGCG delayed the onset of TBARS formation and decreased the extent of TBARS formation compared to when MetHbA was added to the washed muscle (Figure 6B).

**Figure 6:**
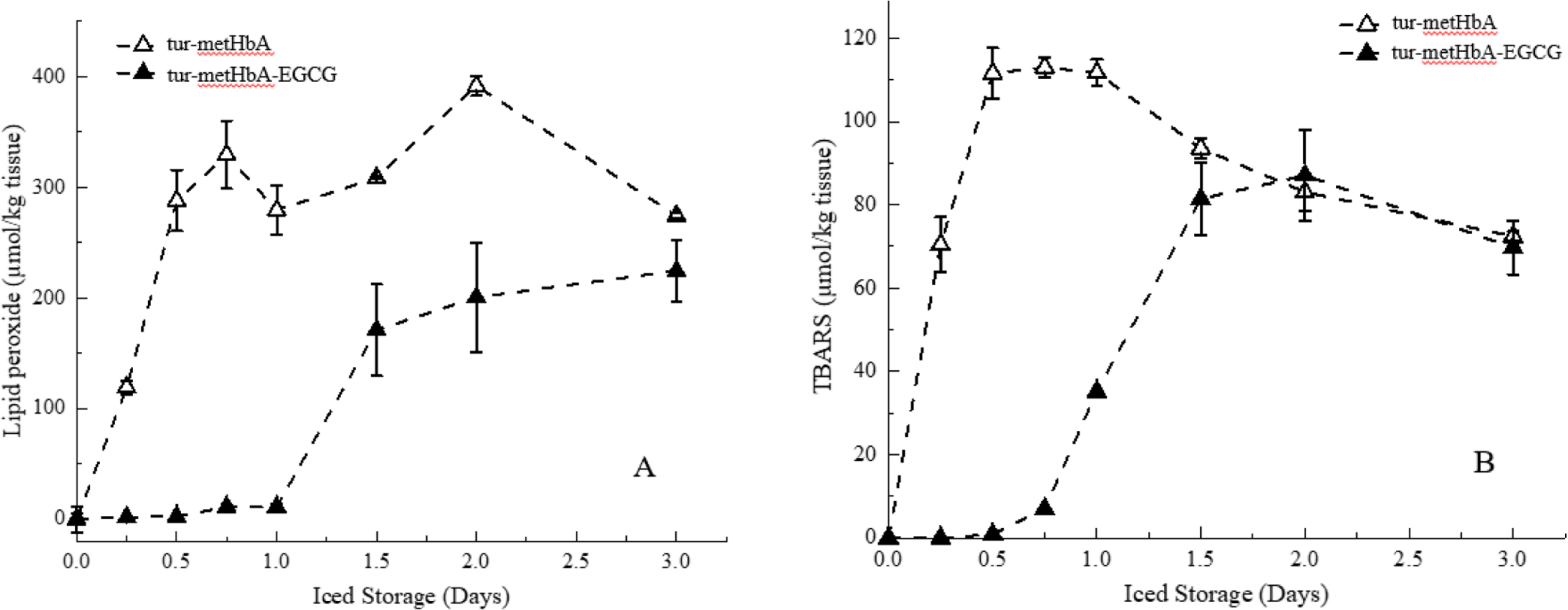
Lipid peroxide (A) and thiobarbituric acid reactive substances (TBARS) values (B) of MetHbA and MetHbA-EGCG adduct in washed muscle during 2 °C storage. pH was 6.3. Hb concentration was 30 μmol/kg washed cod (heme basis).

## Discussion

### Ferryl Suppression

The ability of bound EGCG to reduce H_2_O_2_-mediated ferryl Hb formation (Figure 4C) likely occurs by *indirect* electron transfer noting the distances from the ionizable groups of bound EGCG to the heme iron moieties within hemoglobin. Our crystal structure of Hb-EGCG indicated distances of 11.7–16.5 Å from the nearest phenol/phenolate group of bound EGCG to the nearest heme iron atom (Figure 3C). It might be expected that bound EGCG is too distant from the iron atom of each heme moiety to be able to decrease ferryl Hb formation. At the same time, Mayo et al.^51^ described that electron transfer can take place at distances greater than 10 Å through heme protein interiors. Electron tunneling of 4 to 24 Å has been described in heme proteins.^52^ The ability of covalently bound EGCG to deplete ferryl Hb may partly explain the decreased degree of lipid oxidation incurred by oxyHb-EGCG compared to oxyHb (Figure 5) as well as MetHb-EGCG compared to MetHb (Figure 6).

### Cross-Linking

It was puzzling that protein crystallography showed EGCG covalently bound at αCys130 with no evidence of cross-linking between subunits, whereas SDS-PAGE indicated there was intersubunit cross-linking (Figure 4D). The photo energy present during X-ray exposure and data acquisition of the Hb crystals may have broken a covalent bond in the Hb-EGCG crystal structure. It is also possible that the cross-linked species represents a minor but functionally significant population that did not crystallize. The crystal structure shows the most stable, predominant state in the crystal lattice, while SDS-PAGE may capture the entire distribution in solution. Solution-based size analysis should also be done to further evaluate the Hb cross-linking due to added EGCG, noting SDS-resistant associations can occur.

### Hemin Release Suppression

The ability of EGCG to incur Hb subunit cross-linking may explain the decreased hemin dissociation observed for MetHb-EGCG compared to MetHb alone (Figure 4B). Intersubunit cross-linking is likely to decrease hemin dissociation since monomers release hemin more readily than dimers that in turn release hemin slower than Hb tetramers.^53^ Thus, EGCG-incurring intersubunit cross-linking can decrease Hb subunit formation that leads to higher hemin affinity. Elevated hemin affinity is important in the context of inhibiting lipid oxidation by limiting the release of hemin from the globins into cellular membranes that contain preformed lipid hydroperoxides.^47^ Hemin readily degrades lipid hydroperoxides to free radicals that readily abstract hydrogen atoms from polyunsaturated fatty acids within the phospholipids to propagate extensive lipid oxidation.^54^ It can be noted that MetHb-containing bound EGCG promoted lipid oxidation in washed muscle less effectively than MetHb (Figure 6). This can be attributed to EGCG-induced cross-linking of Hb so as to decrease hemin dissociation.

### Autooxidation Accelerated

Bound EGCG accelerated the conversion of oxyHb to MetHb (Figure 4A). This can be attributed to indirect electron transfer from bound EGCG to liganded O_2_ within the heme crevice of oxyHb. Previously, it was shown that phenol readily reacted with oxyHb to form MetHb and superoxide anion radical.^55^ Galloylated polyphenols similar to EGCG have also been shown to reduce the redox stability of fish Hb.^11^ It should be noted that dithionite was used to prepare reduced Hb and reduced Hb-EGCG. It cannot be ruled out that side reactions from dithionite addition interacted with Hb-EGCG in such a way as to accelerate the onset of MetHb formation. The spectral changes indicative of increased MetHb formation in Hb-EGCG compared to Hb are also shown (Figures S1 and S2).

### Lipid Oxidation Suppression

It can be noted that bound EGCG inhibited MetHb-mediated lipid oxidation (Figure 6) more effectively than oxyHb-mediated lipid oxidation (Figure 5). The ability of bound EGCG to increase MetHb formation (pro-oxidative) and decrease ferryl Hb formation and hemin dissociation from MetHb (antioxidative) likely explains the better ability of bound EGCG to inhibit lipid oxidation due to MetHb compared to that due to oxyHb. In that, bound EGCG facilitates MetHb and superoxide anion radical formation when adding oxyHb-EGCG to washed muscle, whereas when adding MetHbA-EGCG to washed muscle, superoxide anion radical will not be generated due to the absence of liganded O_2_, and only the antioxidative attributes of bound EGCG will be manifested, namely, decreased hemin dissociation and decreased ferryl Hb formation. It was similarly described that the superoxide anion radical released from oxyMb increased the lipid oxidation capacity in a liposome system compared to metMb.^56^

### Increased Negative Charge on Hb

EGCG bound to Hb can either be electrostatically neutral if the phenolates are protonated or negatively charged if some of the phenolate groups are deprotonated. This was investigated by using native electrophoresis. HbA adducted with EGCG migrated further toward the positive pole during native electrophoresis than nonadducted HbA, indicating that EGCG bound to Hb imparted increased negative charge to the heme protein. This may be relevant regarding interactions of Hb with lipids in the insoluble phase of muscle. It was previously shown that the relatively positively charged trout I Hb bound more readily to insoluble components of washed muscle than trout IV Hb that has a relatively negative overall charge.^57^ Thus, it is plausible that covalently bound EGCG makes Hb less oxidative toward lipids by electrostatically reducing the interaction of the heme protein with lipids.

### Other Considerations

It should be emphasized that the turkey Hb-EGCG structure observed in the crystalline state may differ from that in solution. Future work of hydrogen–deuterium exchange mass spectrometry (HDX-MS) and solution NMR/fluorescence quenching experiments should be done to probe EGCG accessibility and conformational dynamics outside the crystalline state. The secondary structure of turkey Hb compared to turkey Hb-EGCG should also be evaluated in solution, noting that noncovalently bound EGCG was described to decrease the α-helicity of bovine Hb.^14^ It should be noted that mammalian and fish Hbs (bovine, porcine, and trout IV) do not contain cysteine at the analogous α-chain site (13th residue of the H-helix). This indicates that EGCG bonding that occurs at αCys130 of turkey HbA will not occur at the analogous site of mammalian and fish Hbs so that our results are not directly translatable to those of mammals and fish. Although our crystallography analysis indicates only αCys130 of turkey HbA was adducted with EGCG, it is possible that other Cys residues of turkey HbA may covalently bind EGCG to some degree, noting that βCys93, αCys104, and βCys112 of human Hb were accessible to p-hydroxymercuribenzoate.^58^ Mutational studies to convert αCys130 to Ala represent an approach to block EGCG binding to gain insight on the oxidative capacity of turkey HbA when added EGCG cannot bind at that site. However, γCys93Ala of human fetal Hb had decreased oxidative stability compared to γCys93.^59^ This presents a complicating factor when interrogating the effect of blocking EGCG bonding to αCys130 of turkey HbA by the site-directed mutagenesis approach.

In conclusion, the pyrogallol unit of EGCG becomes covalently attached to turkey HbA at αCys130 based on the crystal structure. Noting that the mass increase was 454 *m*/*z* based on ESI-MS, it appears EGCG reduced MetHb, causing the formation of the galloyl quinone and the pyrogallol quinone that reacts with αCys130, regenerating the pyrogallol, while the galloyl quinone remains. Hb-EGCG had both pro-oxidative (increased Hb oxidation to MetHb) and antioxidative effects (decreased hemin dissociation and decreased ferryl Hb formation). Decreased hemin dissociation from MetHb appeared related to the ability of added EGCG to incur Hb cross-linking. Phenol/phenolate groups of bound EGCG were greater than 10 Å from the heme-iron moieties, suggesting indirect electron transfer to deplete ferryl Hb and convert oxyHb to MetHb. Bound EGCG more effectively inhibited MetHb-mediated lipid oxidation compared to oxyHb-mediated. This was likely due to liganded O_2_ becoming activated by bound EGCG when assessing oxyHb-EGCG, whereas MetHb-EGCG has no O_2_ ligand. Future work should evaluate electron transfer processes of Hb containing bound EGCG to better understand mechanisms by which bound EGCG increased Hb autoxidation and decreased ferryl Hb formation. Further, the ability of bound EGCG to increase the net negative charge of Hb should be examined in the context of Hb-lipid interactions.

## Supplementary Material



## Notes

Data collection and refinement statistics for the crystal structures of turkey HbA and turkey HbA that contains covalently bound epigallocatechin gallate (EGCG); the PDB IDs for HbA and HbA-EGCG that are 12RM and 12RH, respectively (Supplemental Table 1); spectra changes of HbA and HbA-EGCG during storage as part of the Hb autoxidation measurement (Figures S1 and S2) (PDF)

## #

State Key Laboratory of Advanced Separation Membrane Materials, Zhejiang Key Laboratory of Surface and Interface Science and Engineering for Catalysts, College of Chemical Engineering, Zhejiang University of Technology, Hangzhou 310014, China

## ∇

Food Biotechnology Research Team, National Center for Genetic Engineering and Biotechnology (BIOTEC), 113 Thailand Science Park, Phahonyothin Rd., Khlong Nueng, Khlong Luang, Pathum Thani 12120, Thailand

## Abbreviations

CA: caffeic acid
EGCG: epigallocatechin-3-gallate
ESI-MS: electrospray ionization mass spectrometry
FPLC: fast protein liquid chromatography
HbA: turkey hemoglobin A
HbD: turkey hemoglobin D
MetHb: methemoglobin
SDS-PAGE: sodium dodecyl sulfate polyacrylamide gel electrophoresis
SOD: superoxide dismutase
TBA: 2-thiobarbituric acid
TBARS: thiobarbituric acid reactive substances
TCA: trichloroacetic acid
WCM: washed cod muscle
