# Supplementary material for: Oxidative Characteristics of Turkey Hemoglobin A Containing Covalently Bound Epigallocatechin Gallate
Source: J Agric Food Chem. 2026 Jun 9;74(24):18945–55. doi: 10.1021/acs.jafc.5c17482 (PMC13307362; doi:10.1021/acs.jafc.5c17482)
Supplement: Supplementary file 1 [file jf5c17482_si_001.pdf]

Supporting Information

Oxidative characteristics of turkey hemoglobin A containing covalently bound epigallocatechin gallate

Jie Yin[1], Wenjing Zhang[1,3], Nantawat Tatiyaborworntham[1,4], Craig A. Bingman[2] and Mark P. Richards[1*]

[1] Meat Science and Animal Biologics Discovery, Animal and Dairy Sciences Department, University of Wisconsin-Madison, Madison, WI, 53706, USA

[2] Biochemistry Department, University of Wisconsin-Madison, Madison, WI, 53706, USA

[3] Present address: State Key Laboratory of Advanced Separation Membrane Materials, Zhejiang Key Laboratory of Surface and Interface Science and Engineering for Catalysts, College of Chemical Engineering, Zhejiang University of Technology, Hangzhou 310014, China.

[4] Present address: Food Biotechnology Research Team, National Center for Genetic Engineering and Biotechnology (BIOTEC), 113 Thailand Science Park, Phahonyothin Rd., Khlong Nueng, Khlong Luang, Pathum Thani 12120, Thailand

*Address correspondence to:
M.P. Richards
Meat Science and Animal Biologics Discovery
1933 Observatory Drive, Madison WI, 53706
mprichards@wisc.edu
608-262-1792 (P)

Supplemental Table S1 provides the data collection and refinement statistics for the crystal structures of turkey HbA and turkey HbA that contains covalently bound epigallocatechin gallate (EGCG). The PDB ID for HbA and HbA-EGCG are 12RM and 12RH, respectively.

Supplemental Figure S1 provides optical density spectra changes over time from 500 to 400 nm of HbA and HbA-EGCG,

Supplemental Figure S2 provides optical density spectra changes over time from 700 to 500 nm of HbA and HbA-EGCG.

Table S1. Data collection and refinement statistics.

	HbA	HbA-EGCG
**Wavelength**	0.9793	1.033
**Resolution range**	44.65 - 1.695 (1.73 - 1.69)	39.58 - 1.995 (2.04 - 1.99)
**Space group**	P 42 21 2	P 21 21 21
**Unit cell**	81.31 81.31 106.85 90 90 90	80 81.6 90.52 90 90 90
**Total reflections**	477122 (24829)	540410 (37016)
**Unique reflections**	75946 (3930)	40951 (2823)
**Multiplicity**	6.3 (6.3)	13.2 (13.1)
**Completeness (%)**	99.85 (98.51)	99.44 (97.27)
**Mean I/sigma(I)**	8.31 (0.56)	7.99 (1.15)
**Wilson B-factor**	27.78	32.91
**R-merge**	0.1173 (2.926)	0.1928 (2.215)
**R-meas**	0.1284 (3.195)	0.2007 (2.304)
**R-pim**	0.05161 (1.27)	0.05517 (0.628)
**CC1/2**	0.998 (0.225)	0.998 (0.793)
**CC***	0.999 (0.607)	0.999 (0.941)
**Reflections used in refinement**	40394 (2044)	40813 (2812)
**Reflections used for R-free**	2656 (134)	2014 (140)
**R-work**	0.1837 (0.2795)	0.1964 (0.3914)
**R-free**	0.2170 (0.2842)	0.2391 (0.4503)
**Number of non-hydrogen atoms**	2708	5121

**macromolecules**	2242	4499
**ligands**	88	243
**solvent**	378	379
**Protein residues**	284	568
**RMS(bonds)**	0.007	0.109
**RMS(angles)**	0.74	1.40
**Ramachandran favored (%)**	98.57	98.39
**Ramachandran allowed (%)**	1.43	1.61
**Ramachandran outliers (%)**	0.00	0.00
**Rotamer outliers (%)**	0.83	0.41
**Clashscore**	1.51	0.74
**Average B-factor**	33.12	47.11
**macromolecules**	31.81	47.03
**ligands**	28.29	46.61
**solvent**	42.04	48.41

Statistics for the highest-resolution shell are shown in parentheses.

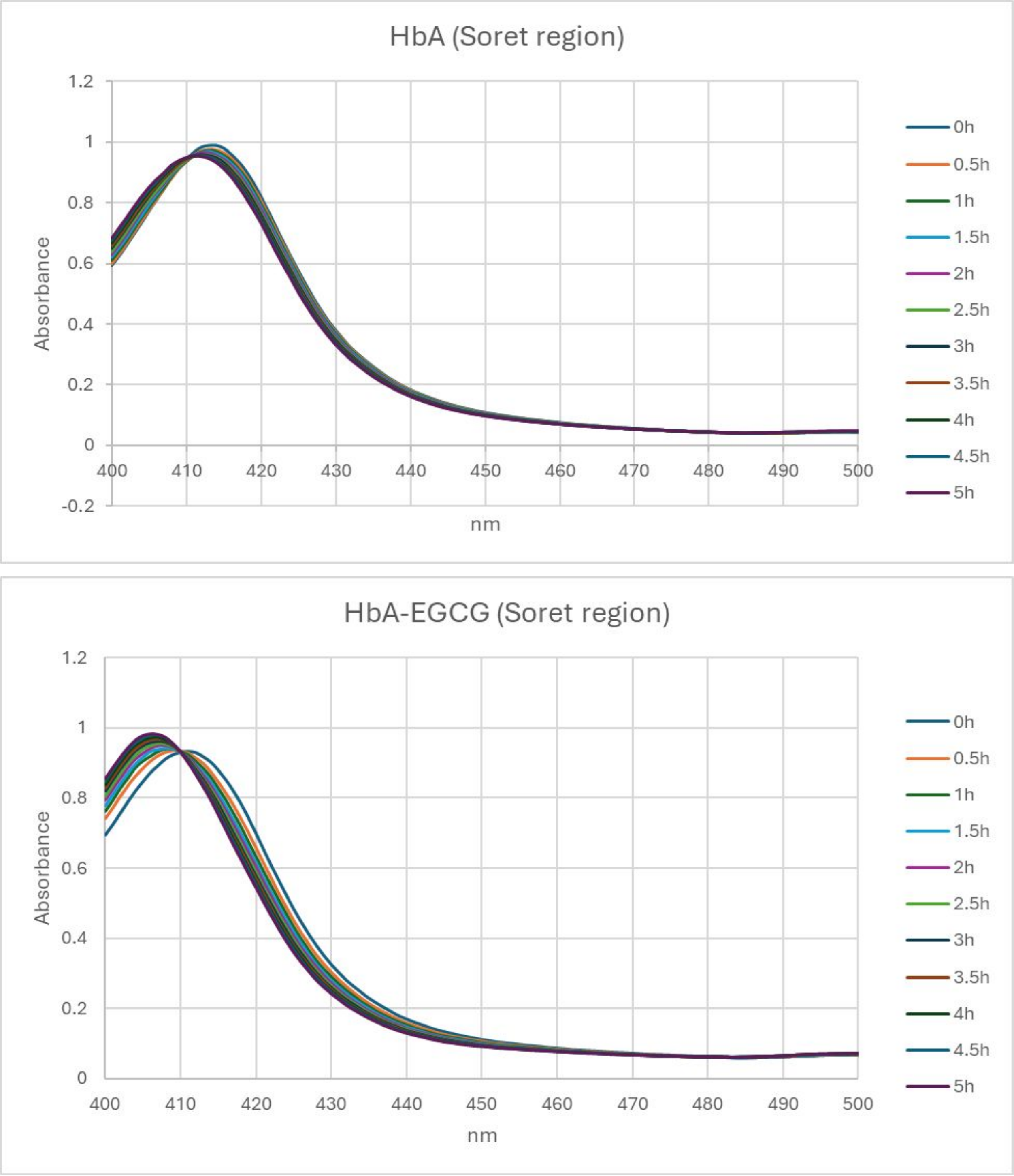

Figure S1. Soret region spectra of Hb and Hb-EGCG during 5 h of storage. The greater shift shift of the peak to lower wavelength of Hb-EGCG is indicative of more rapid conversion of oxyHb to metHb.

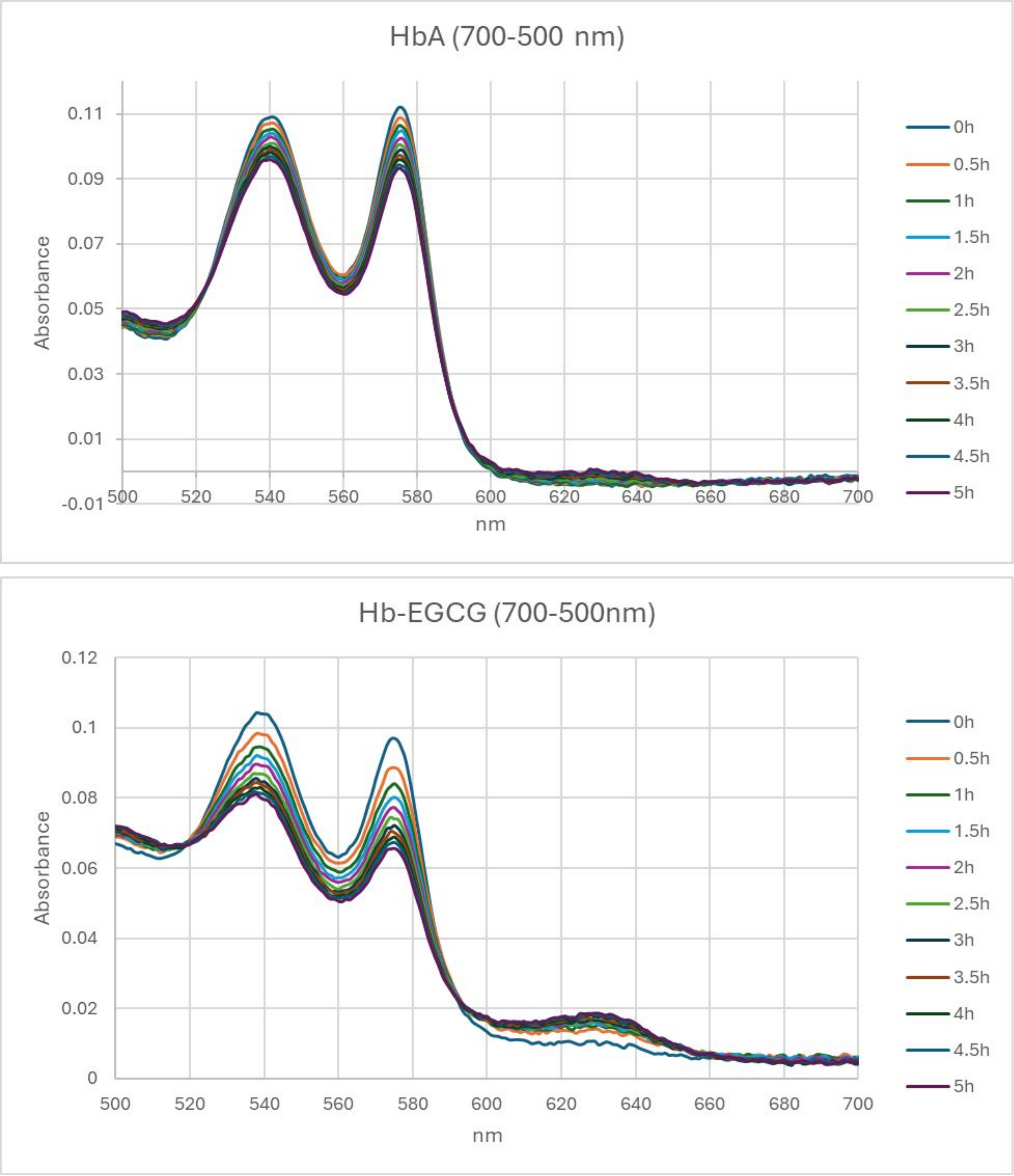

Figure S2. 700 to 500 nm spectra of Hb and Hb-EGCG during 5 h of storage. The greater increase in the peak at 630 nm of Hb-EGCG is indicative of more rapid conversion of oxyHb to metHb.
